# Alterations of intestinal mucosal barrier, cecal microbiota diversity, composition, and metabolites of yellow-feathered broilers under chronic corticosterone-induced stress: a possible mechanism underlying the anti-growth performance and glycolipid metabolism disorder

**DOI:** 10.1128/spectrum.03473-23

**Published:** 2024-03-18

**Authors:** Fei Li, Xinyu Chen, Xingyu Xu, Lijun Wang, Jie Yan, Yichen Yu, Xuemei Shan, Rui Zhang, Hua Xing, Tangjie Zhang, Shifeng Pan

**Affiliations:** 1 College of Veterinary Medicine, Yangzhou University, Yangzhou, Jiangsu, China; 2 Meat Processing Key Laboratory of Sichuan Province, Chengdu University, Chengdu, Sichuan, China; 3 Jiangsu Co-innovation Center for Prevention and Control of Important Animal Infectious Diseases and Zoonoses, Yangzhou University, Yangzhou, Jiangsu, China; 4 Department of Animal Science, Washington State University, Pullman, Washington, USA; 5 Guangling College, Yangzhou University, Yangzhou, Jiangsu, China; University of California, Davis, San Bernardino, California, USA

**Keywords:** chronic corticosterone-induced stress, cecal microbiota, differentially expressed metabolites, intestinal mucosal barrier function, glycolipid metabolism disorder, yellow-feathered broilers

## Abstract

This study aimed to explore alterations in growth performance, glycolipid metabolism disorders, intestinal mucosal barrier, cecal microbiota community, and metabolites in a chronic corticosterone (CORT)-induced stress (CCIS) broiler model. Results showed that compared with control (CON) broilers, in CCIS broilers: (i) the final body weight (BW), BW gain, and average daily gain were significantly reduced. (ii) The glycolipid metabolism disorder and impairement of intestinal immune barrier and physical barrier function were observed. (iii) Diversity and richness of cecal microbiota were obviously increased. From phylum to genus level, the abundances of *Firmicutes* and *Faecalibacterium* were significantly decreased, while the abundances of *Proteobacteria*, *RuminococcaceaeUCG-005*, and *Escherichia coli* (*Shigella*) were significantly increased. Microbial network analysis and function pathways prediction showed that cecal microbiota was mainly concentrated in translation, metabolism, nucleotide metabolism, and endocrine system. (iv) The main differential metabolites identified include steroids and their derivatives, amino acids, fatty acids, and carbohydrates; among which 37 metabolites were significantly upregulated, while 27 metabolites were significantly downregulated. These differential metabolites were mainly enriched in pathways related to steroid hormone biosynthesis and tyrosine metabolism. (v) Correlation between cecal microbiota and glycolipid metabolism indexes showed that BW and total cholesterol (TC) were positively correlated with *Christensenellaceae_R.7_group* and *Escherichia_Shigella*, respectively. Furthermore, the downregulated *Faecalibacterium* and *Christensenellaceae* were negatively correlated with the upregulated differentially expressed metabolites. These findings suggested that CCIS altered cecal microbiota composition and metabolites, which led to glycolipid metabolism disorder and impaired the nutritional metabolism and immune homeostasis, providing a theoretical basis for efforts to eliminate the harm of chronic stress to human health and animal production.

**IMPORTANCE:**

The study aimed to determine the influence of altered intestinal mucosal barrier, cecum flora community, and metabolites on anti-growth performance, glycolipid metabolism disorders of chronic corticosterone (CORT)-induced stress (CCIS) broilers. Compared with control (CON) broilers, in CCIS broilers: (i) anti-growth performance, glycolipid metabolism disorder, and impaired intestinal immune barrier and physical barrier function were observed. (ii) From phylum to genus level, the abundances of *Firmicutes* and *Faecalibacterium* were decreased; whereas, the abundances of *Proteobacteria, RuminococcaceaeUCG-005*, and *Escherichia coli* (*Shigella*) were increased. (iii) Differential metabolites in cecum were mainly enriched in steroid hormone biosynthesis and tyrosine metabolism. (iv) Body weight (BW) and total cholesterol (TC) were positively correlated with *Christensenellaceae*_*R.7*_*group* and *Escherichia_Shigella*, respectively, while downregulated *Faecalibacterium* and *Christensenellaceae* were negatively correlated with upregulated metabolites. Our findings suggest that CCIS induces anti-growth performance and glycolipid metabolism disorder by altering cecum flora and metabolites, providing a theoretical basis for efforts to eliminate the effect of chronic stress on human health and animal production.

## INTRODUCTION

Chronic stress refers to the non-specific systemic reaction that occurs when the body is stimulated by various internal and external negative factors over a long time or frequently (1). It is mainly characterized by the activation of hypothalamic-pituitary-adrenal (HPA) axis and the excessive release of blood adrenocorticotropic hormone (ACTH) and glucocorticoids (GCs), resulting in numerous adverse effects (2–8). So at present, a growing number of evidence have shown that repeated injection of corticosterone (CORT) is an ideal method to establish chronic stress model (9). In animals, chronic stress is one of the most dangerous risk factors that seriously harm health, growth performance, and economic benefits (10–15). Moreover, the pathophysiological reaction to chronic stress exposure has long been recognized as a potential factor of various human diseases including obesity, type 2 diabetes, metabolic syndrome, cardiovascular disease, and cancer (16–19). Therefore, anti-stress research is of great importance to both humans and animals based on adverse consequences of chronic stress on homeostasis maintenance.

Chicken is both a major source of meat for humans and an ideal animal model for studying several human metabolic diseases (20). As a persistent disorder syndrome, chronic stress seriously harms the production performance and immune function of poultry (21). Yellow-feathered broilers are a well-known and widely distributed poultry breed that is produced in numerous provinces of China. They are noted not only for their delicious meat and unique flavor, but also for their high feeding and management requirements, weak physique, and poor stress resistance. It is well known that the intestine is not only the main place for digestion and absorption of nutrients, but also an important organ for secretion and immunity. Furthermore, the intestine can also isolate the internal environment of the body from various pathogenic bacteria, showing a very important defense function. Therefore, intestinal health is very important for poultry performance. In particular, in the context of prohibiting and reducing the use of antibiotics in feed, the risk of intestinal bacterial infection increases rapidly, which seriously harms the healthy development of poultry. A growing number of studies have demonstrated that gut microbiota plays an influential role in nutrient absorption and metabolism, and a clear picture of microbiota succession can enhance the host nutrition and disease resistance (22). Although chronic stress in poultry has been investigated extensively, most studies have primarily focused on metabolic alterations, while the roles of gut microbiota involved are largely unknown. Especially, numerous studies have indicated that the abundance of the host gut microbiota is readily affected by various types of stressors, suggesting that the role of gut microbiota is getting more and more attention in studying chronic stress-related diseases. However, whether chronic corticosterone-induced stress (CCIS) affects nutritional metabolism, immune homeostasis, and glycolipid metabolism disorder by altering the structure of host gut microbiota remains largely unclear.

Intestinal microbiota can be considered as a “metabolic organ” and plays an essential role in the regulation of the host biology. Various physiological functions of the host are mediated by the microbiota or their metabolite structural components, which will also be profoundly affected by host metabolic response (23). Therefore, elucidating the composition and metabolic functions of the microbiota, and the interaction between the microbiota and the host nutrient metabolic/digestive processes, will provide new insights into optimizing the intestinal microecological health and nutritional efficiency of animals. Increasing evidence has proven that intestinal microbiota has a variety of biological functions and is of great importance for the growth and development of broilers (24, 25). It has also been suggested that intestinal microbiota can protect the host from pathogens and enhance host immunity (26–28). It is known that there are a large number of pathogenic bacteria in the intestinal tract, such as *Proteus*, which is the known dominant bacteria in the cecal flora of birds, and its proliferation increases the risk of metabolic diseases, which has been proposed as a microbial feature of intestinal flora imbalance (29, 30). In addition, under exogenous stimulation, intestinal flora may exchange alleles with pathogenic bacteria or integrates the pathogenic genes of several pathogenic bacteria to form new pathogenic bacteria, thus increasing the abundance of pathogenic bacteria and causing numerous diseases (31–33). Therefore, the balance of intestinal microbiota is of great significance to the health and production of broilers (34).

The microbiota has various regulatory functions including host development and nutrition (35), digestive performance (36), intestinal physiology (37), and intestinal immune homeostasis (38). The cecum, the intestinal segment with the greatest microbiota density in broiler chickens (39), can supply more than 10% of the host energy demand. Cecum villi and microvilli transport nutrients such as sugars and amino acids into cells (40). The cecum also contributes to the absorption of water and electrolytes and nitrogenous circulation (41). Cecum microbiota plays several probiotic roles including the secretion of active enzymes to deconstruct non-starch polysaccharides and the fermentation of undigested carbohydrates to produce short-chain fatty acids (SCFAs) (42). Furthermore, the cecum microbiota reduces the viscosity of chyme in the intestinal lumen and provides the host with B vitamins.

The intestinal microbiota in poultry is changing dynamically, and its diversity and richness can be affected by breed, diet, feeding conditions, and the environment (43). Therefore, elucidating the developmental dynamics of the cecal microbiota will help optimize the microbial composition or timing of nutrition intervention. However, there is no general agreement on the impact of chronic stress on the structure of the cecal microbiota and intestinal microecology of broiler chickens in the relevant studies. Furthermore, identifying microbiota biomarkers under chronic stress facilitates the better selection and the use of probiotics in fighting against stress-related diseases. For example, promoting the colonization of SCFAs-producing probiotics may improve the absorption of nutrients, thereby affecting the metabolic capacity of the host (44, 45). Related studies have also attempted to identify intestinal microbiota associated with feed efficiency and phenotypic traits in broiler chickens and laying hens (46, 47). However, the microbiota taxa identified so far have different results and poor phenotypic reproducibility.

Therefore, the current study aimed to analyze the CCIS-induced dynamic changes in cecal microbiota and the differentially expressed metabolites in broiler chickens by using 16S rRNA gene sequencing and a non-targeted metabolomics approach, so as to further investigate the alterations of growth performance, glycolipid metabolism, intestinal morphology, mucosal barrier function, and cecal microbiota under CCIS, as well as to explore the correlation between cecal microbiota and host nutrient metabolism. This study contributes to a better understanding of the gut microbiota in metabolic diseases and leads to the discovery of potential candidates for the development of probiotics for chronic stress prevention in broilers.

## MATERIALS AND METHODS

### Establishment of CCIS model in broilers

CCIS model in broilers was established by subcutaneous injection of 4 mg/kg BW CORT (C2505, Sigma-Aldrich Trading Co. Ltd., China), twice a day (9:00 a.m. and 5:00 p.m.) for a week, while broilers injected with the same volume of normal saline were used as the control (CON) group.

### Experimental animals and sample collection

In the study, a total of 60 3-d-old male yellow-feathered broilers with good health and the same body weight (BW) were randomly assigned to the CON group and the CCIS model group (CORT) with three replicates per group and 10 broilers per replicate. The ambient temperature was controlled at 24 ± 2°C, and the humidity was 60 ± 5%. CORT broilers received subcutaneous injection of CORT (4 mg/kg BW) from 46 d old, twice a day for a week (46–52 d old), while CON broilers received the same volume of normal saline. At the end of the experiment (53 d old), in each replicate, four chickens closest to the average weight were selected for slaughter and sampling (*n* = 12). The production performance, blood biochemical indexes and inflammatory factor content, intestinal morphological structure, cecal microbiota, and metabolites were measured and analyzed. During the whole experiment, continuous light, daily ventilation, and disinfection are used; all broilers had free access to food and water; and both the CON and the CORT broilers were fed with the same basic full-price diet. The BW was weighed and recorded regularly to calculate for the body weight gain (BWG) and the average daily gain (ADG). Feed intake was recorded every day during the whole experiment, and the average daily food intake (ADFI) and the feed conversion rate (F/G) were calculated. Segments of duodenum, jejunum, ileum, and cecum were harvested and rinsed several times with normal saline and were divided into two sections. One section (approximately 1–2 cm) was fixed with 4% formaldehyde-phosphate buffer for intestinal morphology detection, and the other section was immediately frozen in liquid nitrogen for RNA extraction and gene expression analysis. The cecum contents were scraped into 1.5-mL aseptic frozen tubes, which were then frozen in liquid nitrogen and transferred to −80°C for the determination of cecal microbiota and metabolites.

### Measurements in plasma samples

The blood samples (10 mL) were collected into heparinizal test tubes immediately, which were centrifuged at 3,000 *g* for 10 min, then the plasma was collected and stored at −20°C for further analysis. Commercial kits (Nanjing Jiancheng Bioengineering Institute, China) were used to determine the content of lipid metabolism indicators, such as glucose (GLU, F006-1-1), total cholesterol (TC, A111-1-1), triglyceride (TG, A110-1-1), high-density lipoprotein cholesterol (HDLc, A112-1-1), low-density lipoprotein cholesterol (LDLc, A113-1-1), and liver injury indicators, such as total protein (TP, A045-2-2), albumin (ALB, A028-2-1), globulin (GLB, H106-1-1), alkaline phosphatase (ALP, A059-2-2), alanine aminotransferase (ALT, C009-2-1), and aspartate aminotransferase (AST, C010-2-1).

### Intestinal morphology

Intestinal tissue sections fixed and preserved by paraformaldehyde were prepared by the standard paraffin embedding technique. The specimens were sectioned at 5-µm thickness and stained with hematoxylin/eosin (H&E). Under 200× microscope, both villus height (VH) and crypt depth (CD) were measured, and villus surface area (VSA) and the ratio of VH to CD (VCR) were calculated. At least 10 intact, well-oriented crypts and villi should be measured in each broiler.

### RNA isolation and qRT-PCR

Total RNA of the cecum sample was extracted with Trizol reagent (Invitrogen Life Technologies, Carlsbad, CA, USA). The integrity of total RNA was checked on denaturing formaldehyde agarose gel, and the concentration of RNA was quantified by Nanodrop 2000 (Thermo Fisher Scientific) spectrophotometer. All primers used in the study were synthesized by Genewiz, Inc (Suzhou, China) and are listed in Table 1. GAPDH (glyceraldehyde-3-phosphate dehydrogenase) was used as a loading control. The relative level of gene expression was calculated in accordance with the standard procedure 2-Ct. All assays were performed at least three times.

**TABLE 1:** Primers used in the present study

Name	Sequence
*TNF-α* (*NM_204267.2*)	F: CGTGGAGACTCAACAGAAGG R: GCAGCGTGTCCAGTTCGCGG
*IFN* (*NM_205427.1*)	F: CTCGCAACCTTCACCTCACCATC
	R: CAGGAACCAGGCACGAGCTTG
*TLR4* (*NM_0011030693.2*)	F: TGAAAGAGCTGGTGGAACCC
	R: CCAGGACCGAGCAATGTCAA
*IL-4* (*NM_001007079.2*)	F: AGCCAGCACTGCCACAAGAAC
	R: CGTGGGACATGGTGCCTTGAG
*MUC2* (*NM_001318434.1*)	F: GTGAAGACCCTGATGAAA
	R: GTGAACACTGGCGAGAAT
*LEAP2* (*NM_001001606.1*)	F: ACCCTCGGCTGTCAACTTCTT
	R: TCCTCCTTGGTGCTAATCTCGT
*Claudin-1* (*NM_001013611.2*)	F: CTGATTGCTTCCAACCAG
	R: CAGGTCAAACAGAGGTACAAG
*TFF2* (*XM_040659712.2*)	F: CCCTGCTGATCCTCGTAT
	R: GCTGTTATTTCCCAGTTGA
*GAPDH* (*NM_204305.2*)	F: GAGGGTAGTGAAGGCTGCTG
	R: CGCATCAAAGGTGGAGGAAT

### DNA extraction from cecum contents and 16S rRNA sequencing

Genomic DNA was extracted from the cecum contents of three 53-d-old broilers in both the CON and the CCIS groups, which was then amplified based on the V3-V4 hypervariable region of 16S rRNA. The genomic DNA was extracted by CATB (Cetyltrimethylammonium Bromide) method, and the purity and concentration of DNA were detected by agarose gel electrophoresis. Appropriate amount of DNA samples was taken into centrifuge tube and diluted with sterile water to 1 ng/µL as the template. According to the V3-V4 region of the 16S rRNA gene of the intestinal flora, the specific primers (341F 5′-CCTACGGGRSGCAGCAG-3′ and 806R 5′-GGACTACVGGGTATCTAATC-3′) with barcode were selected. PCR amplification was performed using Phusion High-Fidelity PCR Master Mix with GC Buffer (New England Biolabs, Ipswich, MA, USA) as well as high-efficient and high-fidelity enzymes. PCR was carried out in a 30-µL system consisting of 15-µL Phusion Master Mix (2×), 3 µL of each primer (2 µM), 10 ng gDNA (1 ng/µL), and 2-µL double-distilled water. The PCR procedure was listed as follows: pre-denaturalized for 5 min at 95°C, then 30 cycles of 10 s at 95°C for template denaturation, 30 s at 95°C for primer annealing, 30 s at 72°C for primer extension, and final extension at 72°C for 7 min, followed by cooling to 4°C. The PCR products were detected by 2% agarose gel electrophoresis, which were then recovered by GeneJET kit. The library was constructed using NEBNext Ultra DNA Library Prep Kit for Illumina library building kit. The Illumina Hiseq 4000 platform (Novogene, Beijing, China) was then used for superior sequencing.

### Bioinformatics analysis

The original data (raw data) obtained by 16S rRNA sequencing were stitched and filtered to obtain effective data (clean data). Based on the valid data, QIIME (Version1.9.1) software was used to divide the sequence into operational taxonomic units (OTUs) according to 97% sequence similarity. The sequence with the highest abundance in each OTU was selected as the representative sequence of OTU, and the dilution curve was drawn. According to the OTU abundance matrix, the number of common OTU was calculated by R software (Version3.0.3) and was presented directly by Venn diagram. Chao1, Shannon, Simpson, and ACE indices were all calculated by QIIME software to obtain the information of species richness, evenness, and density in the samples. Both principal component analysis (PCA) and principal coordinate analysis (PCoA) were carried out on the composition and structure of genus-level community by R software, and the natural distribution characteristics among samples were described by two-dimensional images. The statistical algorithm of Metastats in Mothur software was used to determine the sequence quantity (relative abundance) difference of each classification at the phylum and genus level between the CON and CCIS broilers. Linear discriminant analysis Effect Size (LEfSe) analysis was used to screen species with significant differences between the CON and CCIS groups, which can be used to identify the characteristics of different abundance and associated categories. FAPROTAX and Tax4Fun annotation tools were used to predict the functions and metabolic genes of the microbial communities.

### Non-targeted metabolomics analysis

Liquid chromatography combined with mass spectrometry (LC-MS) technology was used for non-targeted metabolomics research; the procedure included sample collection and metabolite extraction, LC-MS detection, metabolic stability quantitative analysis, as well as the data analysis. Three samples of cecum contents from both the CON broilers and the CCIS broilers were placed in six EP tubes. After adding 300 µL of 80% methanol solution, all tubes were put into liquid nitrogen for quick freezing for 5 min. Then, after melting on the ice, the tubes were vortexed for 30 s, followed by ultrasonic treatment for 6 min. The ultrasonic liquid was centrifuged at 5,000 rpm for 1 min at 4°C, then the supernatant was taken into a new centrifuge tube and was frozen into freeze-dried powder, which was then analyzed by LC-MS. In addition, samples were taken from each experimental sample in equal volume and were mixed as quality control (QC). After importing the MS detected raw file (.Raw) into the Compound Discoverer 3.1 software, the qualitative and quantitative data of metabolites were obtained by processing the spectra and searching the database, which were then controlled to ensure the accuracy and reliability of the data. The screening of differential metabolites mainly refers to three parameters: VIP, FC, and *P* value. VIP refers to the variable importance in the projection of the first principal component of the partial least squares discriminant analysis (PLS-DA) model (48), and VIP value indicates the contribution of metabolites to grouping. FC refers to fold change, which is the ratio of the mean of all biological repeated quantitative values for each metabolite in the comparison group. *P* value is calculated by *t*-test and represents the level of significance of the difference. Thresholds were set to VIP > 1.0, FC > 1.2 or FC < 0.833, and *P* value < 0.05 to screen for differential metabolites (48–50). Multivariate statistical analysis of metabolites, including PCA and PLS-DA, was performed to reveal differences in metabolic patterns among the groups. Hierarchical clustering analysis (HCA) and metabolite-metabolite correlation analysis (MMCA) were used to reveal the relationships among samples and metabolites. Finally, the biological significance of metabolites is explained by functional analysis of metabolic pathways.

### Statistical analysis

All data were expressed as mean ± standard error (SE) of the mean. Statistical Program for Social Sciences (SPSS) software 20.0 for Windows (SPSS Inc., Chicago, IL, USA) was used to carry out the statistical analyses. The differences were tested with the one-way analysis of variance (ANOVA), followed by Duncan’s multiple comparisons test. A *P* value of less than 0.05 was considered to be statistically significant. All experiments were repeated at least three times.

## RESULTS

### Effects of CCIS on BW and feed intake of broilers

**Fig 1:**
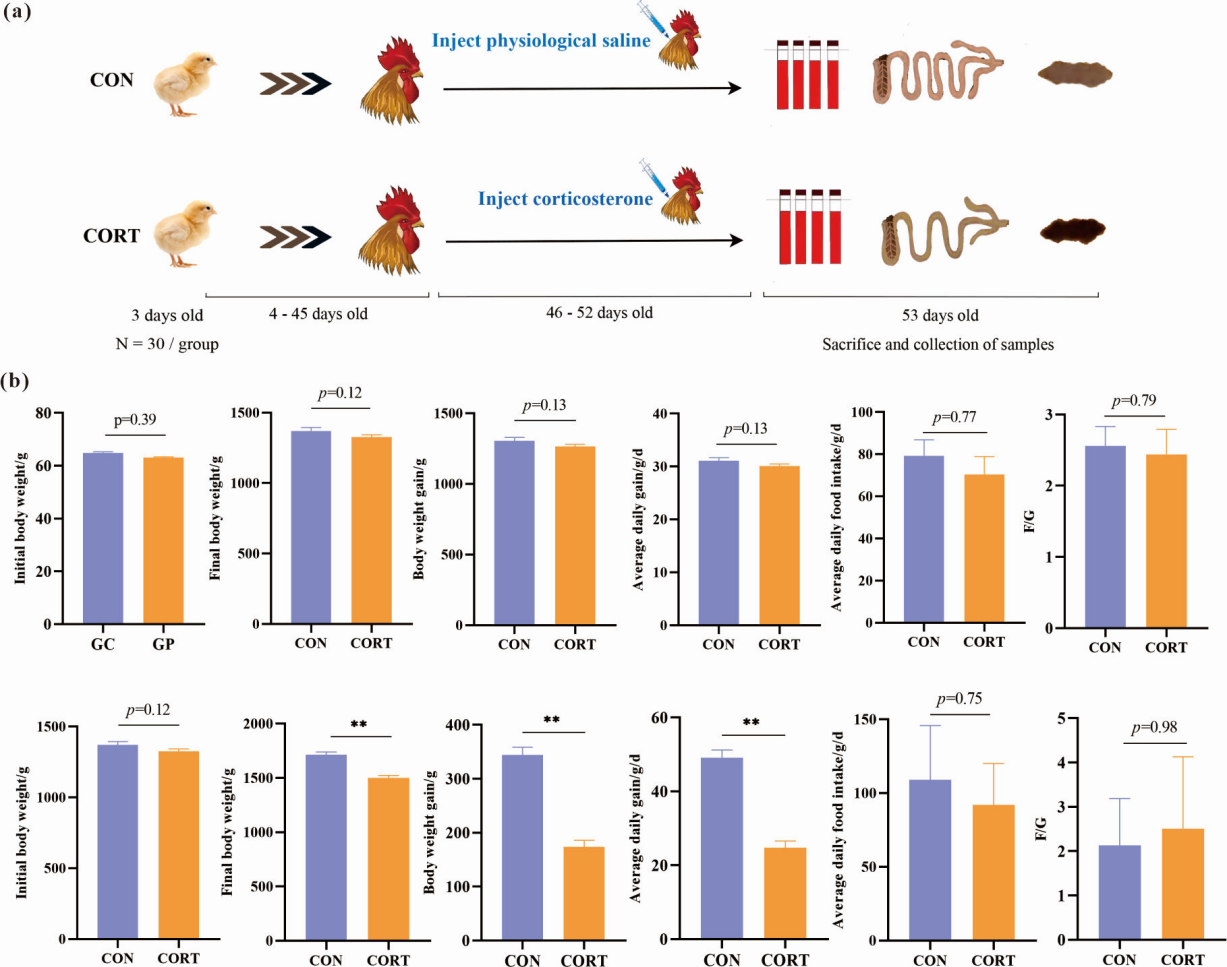
Effects of CCIS on BW and feed intake. (**a**) The experiment-timeline design. (**b**) IBW, FBW, BWG, ADG, ADFI, and F/G before and after CORT injection. All values are presented as means ± SEM, *n* = 30/group. ^**^P ＜ 0.01 vs CON broilers.

As shown in the experimental timeline design (Fig. 1a), the BW of the CON and CCIS broilers were weighed before CORT injection (4–45 d) and after CORT injection (46–52 d). The results showed that there was no significant difference in the initial body weight (IBW), final body weight (FBW), BWG, ADG, ADFI, and the F/G between the CON and CCIS broilers before CORT injection. However, after CORT injection, compared with the CON broilers, although the ADFI and F/G had no significant difference, the FBW, BWG, and ADG were all significantly reduced (Fig. 1b).

### Effects of CCIS on glycolipid metabolism and liver injury indexes in broilers

**Fig 2:**
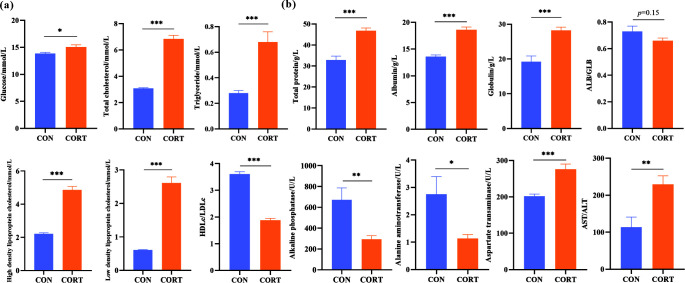
Effects of CCIS on glycolipid metabolism and liver injury indexes. (**a**) The plasma contents of GLU, TC, TG, HDLc, and LDLc, and the ratio of HDLc/LDLc. (**b**) The plasma contents of TP, ALB, GLB, ALP, and ALT. All values are presented as means ± SEM, *n* = 30/group. ^*^P ＜ 0.05, ^**^P ＜ 0.01, ^***^P ＜ 0.001 vs CON broilers.

In order to determine the role of CCIS in regulating glycolipid metabolism, the changes of glycolipid metabolism-related indexes were detected after CORT injection. The results showed that plasma GLU (*P* < 0.05), TC (*P* < 0.001), TG (*P* < 0.001), HDLc (*P* < 0.001), and LDLc (*P* < 0.001) were all significantly increased in the CCIS broilers compared with the CON broilers, while the HDLc/LDLc ratio was significantly decreased (*P* < 0.001) (Fig. 2a), suggesting that CCIS resulted in an increase in the incidence of glycolipid metabolism disorders.

As a key metabolic organ, the liver plays a vital role in various aspects of glycolipid metabolism, and glycolipid metabolism disorders may increase the risk of liver damage. Therefore, we speculate that glycolipid metabolism disorders caused by CCIS may be related to liver damage. The results showed that the contents of TP, ALB, GLB, and AST were all significantly increased (*P* < 0.001), while both ALP (*P* < 0.01) and ALT (*P* < 0.05) were significantly decreased. Furthermore, the AST/ALT ratio was obviously increased (*P* < 0.01), suggesting that CCIS resulted in liver damage to some extent, which might be closely related to glycolipid metabolism disorders in broilers (Fig. 2b).

### Effect of CCIS on intestinal immune barrier and physical barrier function in broilers

**Fig 3:**
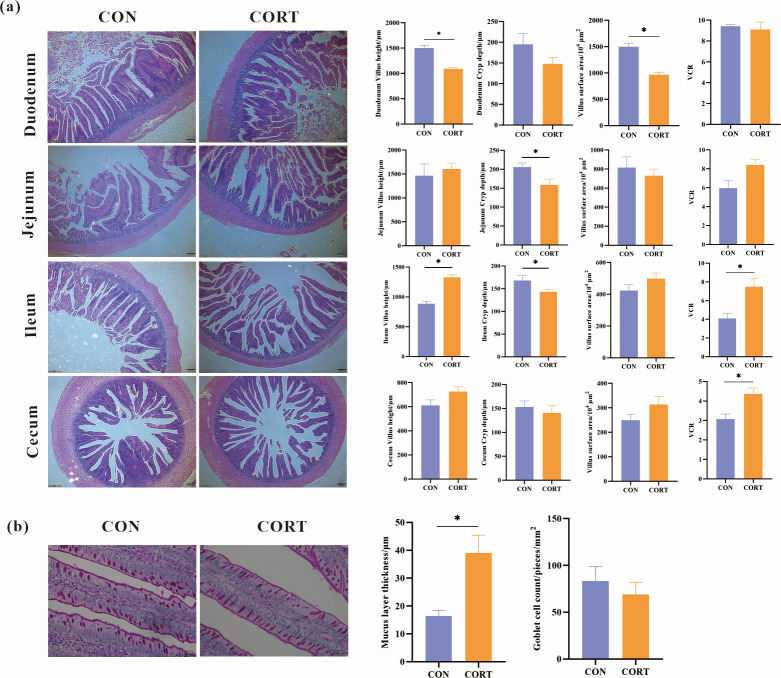
Effects of CCIS on intestinal mucosal barrier function. (**a**) Effects of CCIS on the morphology of the duodenum, jejunum, ileum, and cecum after H&E staining (200×), and the statistical results of VH, CD, the ratio between VH and CD of the duodenum, jejunum, ileum, and cecum. (**b**) Effects of CCIS on the thickness of jejunal mucus layer and the number of goblet cells per unit villi after PAS staining (20×). All values are presented as means ± SEM, *n* = 3/group. ^*^P ＜ 0.05 vs CON broilers.

The intestinal barrier integrity is essential for the absorption of nutrients and health in both humans and animals. Dysfunction of the mucosal barrier is associated with increased gut permeability and development of multiple gastrointestinal diseases. To further determine the effect of CCIS on intestinal integrity and mucosal barrier function, histological and histometric analyses were performed for the H&E-stained sections of duodenum, jejunum, ileum, and cecum, as well as the PAS (Periodic Acid-Schiff)-stained sections of jejunum. The VH, CD, VSA, and VCR were observed and measured under microscope (200×). The results showed that both the VH and the VSA of duodenum were significantly reduced (*P* < 0.05). However, the VH of ileum and the VCR of ileum and cecum were both significantly increased (*P* < 0.05), while the CD of jejunum and ileum were both significantly reduced (*P* < 0.05) (Fig. 3a).

Furthermore, the PAS-stained sections of jejunum were observed under microscope (20×); the mucus layer thickness of jejunum was measured and the number of mucus-containing goblet cells per unit villi was counted. Compared with the CON broilers, although the number of goblet cells showed no significant change in the CCIS broilers, the thickness of mucus layer of jejunum was significantly increased (*P* < 0.05), suggesting that CCIS affected the intestinal development and function of broilers by changing the intestinal morphology (Fig. 3b).

**Fig 4:**
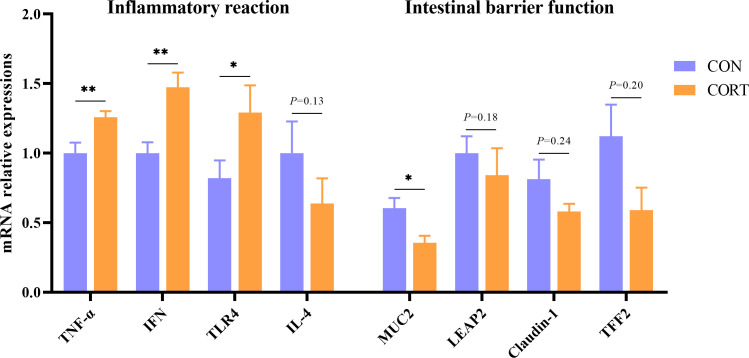
Effects of CCIS on intestinal immune barrier and physical barrier function-related gene expression. (**a**) Inflammatory reaction-related gene expression. (**b**) Intestinal barrier function-related gene expression. All values are presented as means ± SEM, *n* = 12/group. ^**^P ＜ 0.01, ^*^P ＜ 0.05 vs CON broilers.

PCR results showed that the expression of inflammatory reaction-related genes TNF-α, IFN, and TLR-4 was significantly increased, while IL-4 expression has no change. Furthermore, although LEAP2, Claudin-1, and TFF2 expression did not change, the expression of MUC-2 was significantly decreased, demonstrating that CCIS significantly impaired the intestinal immune barrier and physical barrier function in broilers (Fig. 4).

### Analysis of alpha diversity of the cecum microbiota by 16S rRNA sequencing

**Fig 5:**
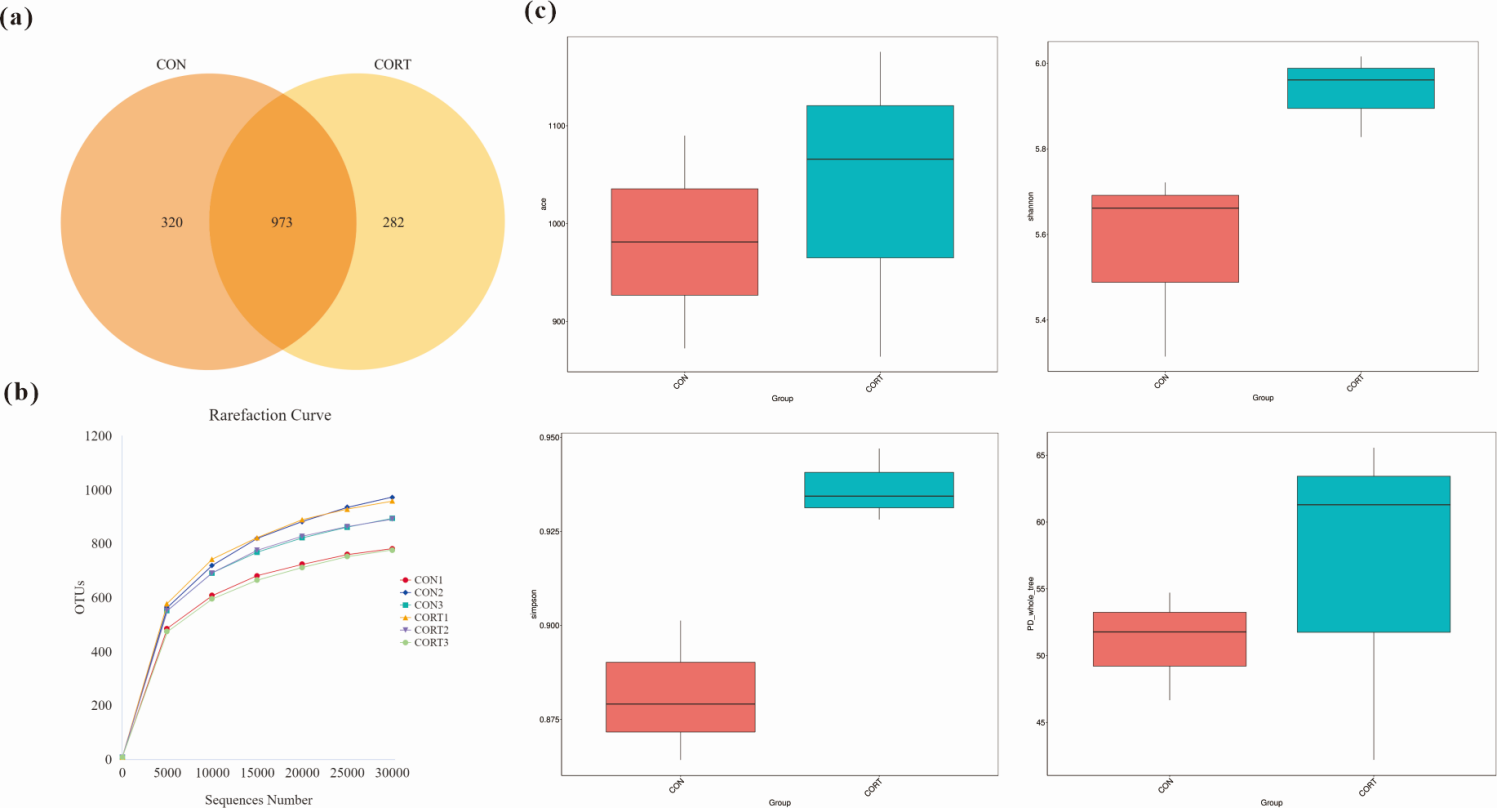
Analysis of alpha diversity of the cecum microbiota by 16S rRNA sequencing. (**a**) The OTUs level Wayne diagram. (**b**) Rarefaction curve. (**c**) Alpha diversity of the cecum microbiota. ACE index, species richness of cecum microbiota. Shannon index, Simpson diversity index, species diversity of cecum microbiota. PD, pedigree diversity. All values are presented as means ± SEM, *n* = 3/group.

A total of 1,575 OTUs were obtained by OTU clustering. Among them, there were 320 OTUs in the CON broilers, 282 OTUs in the CCIS broilers, and 973 OTUs in both groups (Fig. 5a), which could be divided into 32 phyla, 58 classes, 108 orders, 156 families, 281 genera, and 120 species. The rarefaction curves of all samples were obtained based on the OTUs of the CON group and the CCIS group. The results showed that with the increase of the amount of sequencing, the linearity of the rarefaction curve of each sample seemed to be smooth, and the species of cecum microbiota was close to the saturation stage, indicating that the cecum microbiota had high species richness and wide coverage, and the amount of sequencing data was fully satisfactory to characterize the diversity and composition of the cecum microbiota (Fig. 5b).

The ACE index, Shannon diversity index, Simpson diversity index, and PD_Whole_Tree index of bacterial species were used to determine the alpha diversity, so as to investigate the effects of CCIS on the diversity and richness of the cecum microbiota. Compared with the CON group, the ACE index, Shannon index, Simpson index, and PD_Whole_Tree index of the CCIS group were all significantly increased. Furthermore, both the microbial diversity and richness were obviously increased, indicating that CCIS changed the diversity and richness of the cecum microbiota (Fig. 5c) and that CCIS impaired intestinal development and function by modulating the microbial community.

### Effect of CCIS on species composition and changes of cecum microbiota in broilers

#### Phylum level

**Fig 6:**
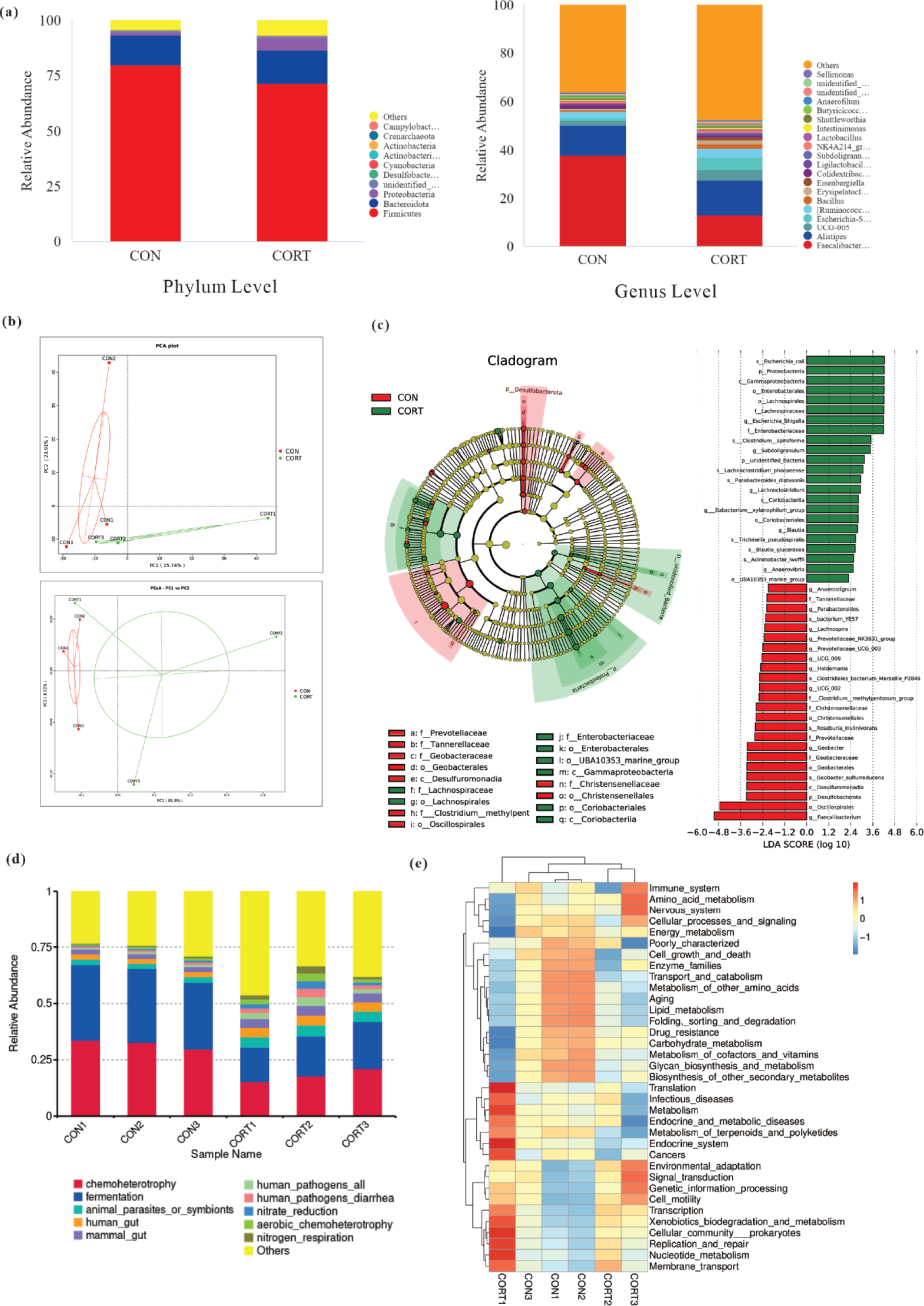
Effects of CCIS on species composition, structure, and function of cecum microbiota. (**a**) Composition of cecum microbiota at the phylum level and genus level. (**b**) Genus-level PCA and OTU-level PCoA based on unweighted UniFrac distances. (**c**) Branching map of LEfSe analysis showing the microbial structure that differed most between the CON and CORT broilers. (**d**) Species classification bar chart between the CON and CORT broilers. (**e**) KEGG metabolic pathway heat map.

At the phylum level, the dominant phyla of the CON group and the CCIS group were *Firmicutes*, *Bacteroidota*, and *Proteobacteria*, accounting for more than 92% of the total phyla. However, compared with the CON broilers, the proportion of *Firmicutes* in the CCIS broilers was significantly decreased, while the proportion of *Proteobacteria* was significantly increased (Fig. 6a), indicating that CCIS could significantly decrease the abundance of *Firmicutes* and increase the abundance of *Proteobacteria* in the cecum microbiota.

#### Genus level

At the genus level, both the *Faecalibacterium* and *Alistips* showed higher abundance in each detected sample. Compared with the CON group, the abundance of *Faecalibacterium* was significantly decreased in the CCIS group, while the abundance of *UCG-005* and *Escherichia_Shigella* was significantly increased (Fig. 6a), suggesting that CCIS could increase the abundance of pathogenic bacteria in the cecum microbiota.

### Effect of CCIS on cecum microbiota composition and community structure differences

#### PCoA and PCA

The structural similarity of microbial community was observed using PCoA and PCA plots, based on the Bray-Curtis distance of OTUs between the CON group and the CCIS group. The results showed that all samples in the CON group and the CCIS group were far apart, suggesting that there were significant differences in cecum microbiota between the CON and CCIS broilers (Fig. 6b).

#### LEfSe analysis

The difference of microbial community composition between the CON group and the CCIS group was analyzed by linear discriminant analysis (LDA) effect sizes (LEfSe). The cladistic diagram showed the most abundant microbial species in the CON group and the CCIS group at the taxonomic levels of phylum, class, order, family, and genus. Forty-seven different species were found between the CON broilers and the CCIS broilers, among which 24 were enriched in the CON broilers and 23 in the CCIS broilers. LEfSe analysis results showed that 24 bacterial species were abundant in the CON broilers, including *g_Lachnospira*,*g_Anaerostignum*,*g_UCG_009*,*f_Tannerellaceae*,*s_Clostridiales_bacterium_Marseille_P2846*,*g_Parabacteroides*,*s_bacterium_YE57*,*g_Prevotellaceae_NK3B31_group*,*g_Prevotellaceae_UCG_003*,*g_UCG_002*,*f_Christensenellaceae*,*o_Christensenellales*,*f_Clostridium_methylpentosum_group*,*f_Prevotellaceae*,*g_Holdemania*,*s_Roseburia_inulinivorans*,*f_Geobacteraceae*,*g_Geobacter*,*p_Desulfobacterota*,*c_Desulfuromonadia*,*s_Geobacter_sulfurreducens*,*o_Geobacterales*,*o_Oscillospirales and g_Faecalibacterium*, while 23 bacteria were enriched in CORT broilers, including *f_Lachnospiraceae*,*o_Lachnospirales p_Proteobacteria*,*c_Gammaproteobacteria*,*f_Enterobacteriaceae*,*s_Escherichia_coli*,*o_Enterobacterales*,*g_Escherichia_Shigella*,*g_Subdoligranulum*,*s_Clostridium_spiroforme*,*s_Parabacteroides_distasonis*,*p_unidentified_Bacteria*,*s_Lachnoclostridium_phocaeense*,*g_Lachnoclostridium*,*s_Acinetobacter_lwoffii*,*g_Eubacterium_xylanophilum_group*,*g_Blautia*,*s_Blautia_glucerasea*,*g_Anaerovibrio*,*s_Trichinella_pseudospiralis*,*o_Coriobacteriales*,*e_Coriobacteriia* and *o_UBA10353_marine_group* (Fig. 6c), suggesting that CCIS resulted in a significantly different microbial diversity.

### Effect of CCIS on cecum microbiota function in broilers

The functional classification of changed cecum microbiota was carried out by using FAPROTAX software. Results showed that compared with the CON group, the abundance of heterotrophic and fermented flora in the CCIS group was significantly decreased, while the abundance of human pathogens, diarrhea pathogens, and nitrate reduction flora was increased significantly (Fig. 6d).

Furthermore, the Tax4Fun method was used to predict the gene function of the differentially expressed cecum microbiota. Results showed that the cecum microflora of the CCIS broilers was enriched in translation, metabolism, nucleotide metabolism, endocrine system, and other gene functions. However, the abundance of genes involved in immune system, energy metabolism, drug resistance, glucose metabolism, and other functions was relative low, showing a negative correlation (Fig. 6e).

### Multivariate statistical analysis of cecum microbial metabolites in broilers

**Fig 7:**
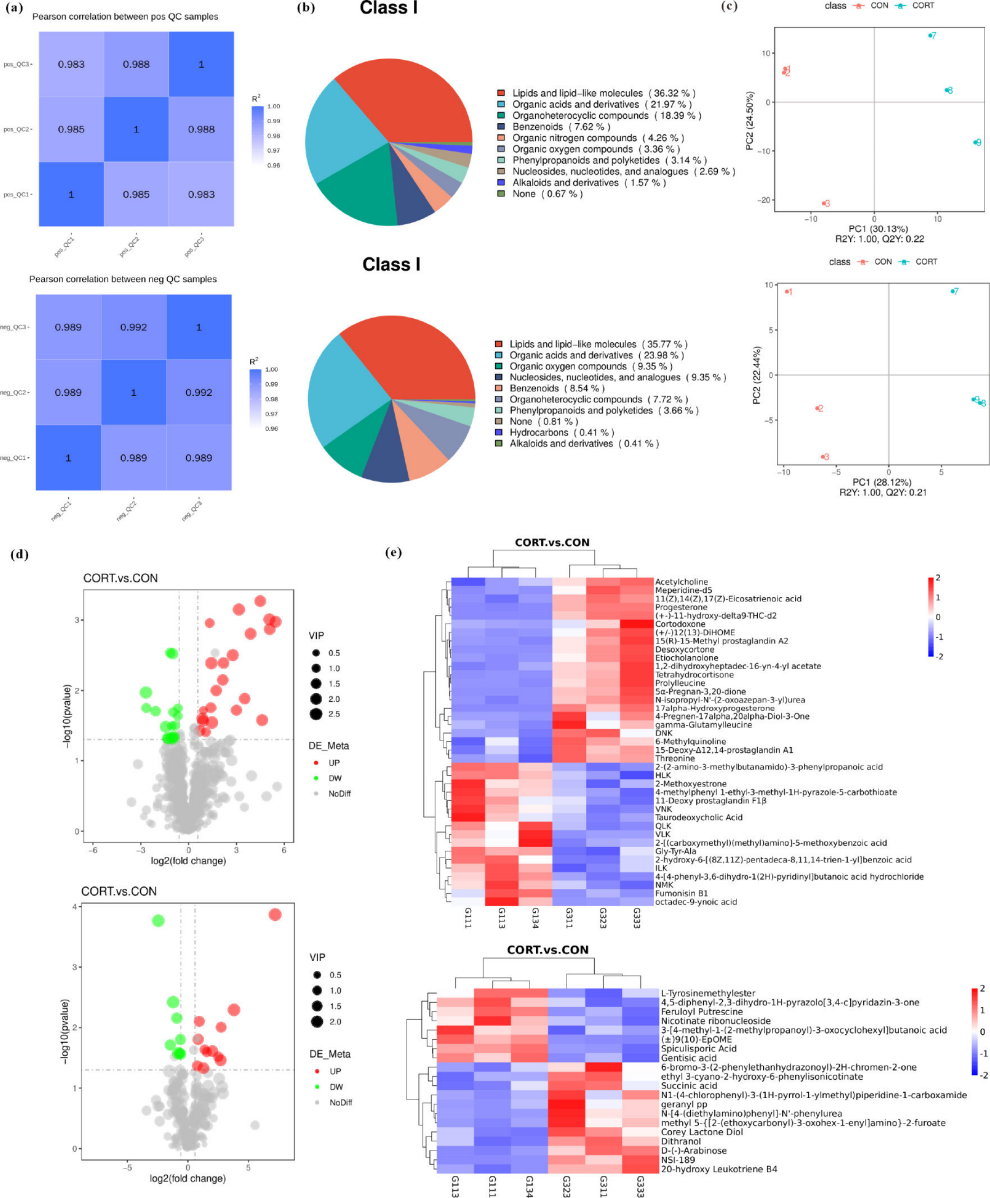
Multivariate statistical analysis of metabolite data of cecum microbial samples. (**a**) QC sample correlation analysis. (**b**) Metabolite class pie chart (**c**) PCA for the cecum microbial metabolism samples. (**d**) Differential metabolite volcano plot. (**e**) Differential metabolite cluster heat map between the CON and CORT broilers.

The 16S rRNA sequencing results above showed significant structural changes of cecum microbiota, but its function was only preliminarily predicted and described. Cecum microbiota is always involved in the regulation of the host metabolism, so the metabolite of the cecum microbiota is regarded as a functional readout of the cecum microbiota. In the study, untargeted metabolomics by using the LC-MS/MS method was applied for metabolite analysis. The results of correlation analysis of the QC samples showed that correlation coefficients were all greater than 0.98 and close to 1, suggesting that the detection system has good stability and high sensitivity (Fig. 7a). As a result, a total of 710 positive-ion-mode metabolites and 314 negative-ion-mode metabolites were identified, among which lipids and lipid-like molecules, organic acids and their derivatives, organic oxides, and organic heterocyclic compound were the major constituents (Fig. 7b).

### Metabolite analysis of cecum microbial samples in broilers

PCA results showed that the samples detected were well dispersed and that significant differences were observed between the CON broilers and the CCIS broilers (Fig. 7c). Based on the screening conditions (VIP > 1.0, FC > 1.2 or FC < 0.833, and *P* value < 0.05) of intestinal microbial differential metabolites, the top 20 differential metabolites were displayed in a chord plot according to the correlation coefficient of differential metabolites, and the *P* values are sorted from small to large. The differential metabolite volcano plot showed that 43 metabolites under positive-ion pattern differed significantly between the CON group and the CCIS group, among which progesterone, 17α-hydroxyprogesterone, deoxycorticosterone, proline leucine, taurine cholic acid, and 24 other metabolites were significantly upregulated, while butyrate, fumonisin B1, and 19 other metabolites were significantly downregulated. In addition, there were 21 metabolites under negative-ion pattern that significantly changed, among which 13 metabolites, such as phosphate, arabinose, methionine, and succinic acid, were significantly upregulated, while eight metabolites of capsaicin, gentisic acid, and nicotinic ribonucleotide were significantly downregulated (Fig. 7d and e). Hierarchical clustering analysis showed the clustering of metabolite content between the two groups in the positive and negative-ion modes, which means the samples in each group were consistent, and most of the metabolite content was higher in the CCIS group compared with the CON group (Fig. 7e).

### Metabolic pathway analysis of different metabolites in cecum microbial samples in broilers

Based on the above data, a systematic pathway annotation was further performed for all identified metabolites, and the KEGG enrichment analyses were performed for all differential metabolites. At the secondary classification level, 18 metabolic pathways were obtained in the KEGG pathway database, among which the most representative functional categories were associated with global and overview maps, lipid metabolism, amino acid metabolism, cofactor and vitamin metabolism, carbohydrate metabolism, and nucleotide metabolism.

**Fig 8:**
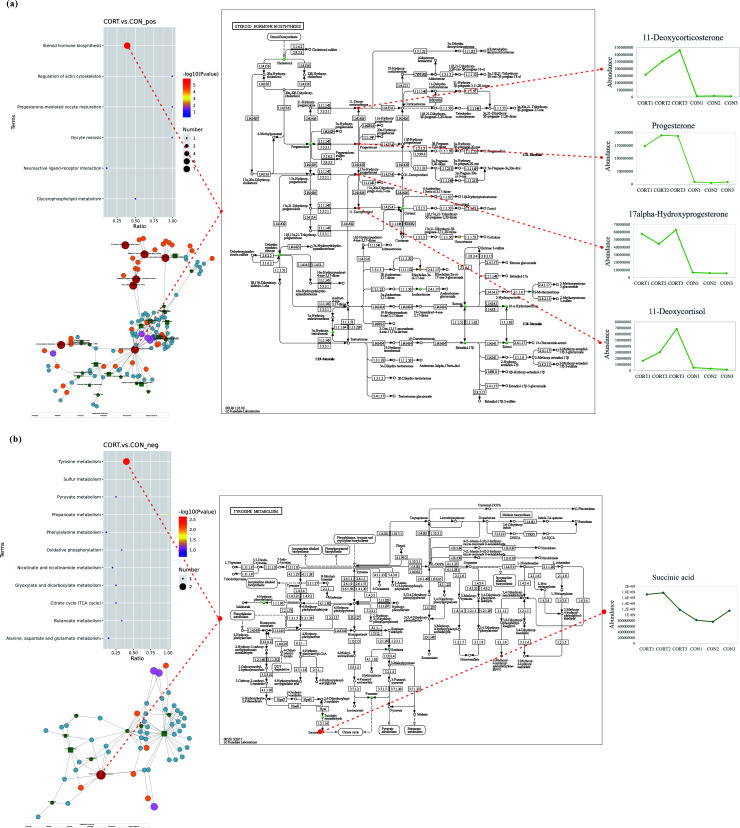
Metabolic pathway analysis of different metabolites in cecum microbial samples. (**a**) The most different cecal metabolites in positive-ion mode. (**b**) Metabolites that differed the most in negative-ion mode.

The KEGG enrichment pathway was mapped by using *P* value < 0.05 and FDR < 0.1 as screening conditions (Fig. 8). Steroid hormone biosynthesis is the largest number of quantified metabolites in the positive-ion model, while in the negative-ion model, three different metabolite pathways were identified, including tyrosine metabolism, sulfur metabolism, and propanoate metabolism, among which tyrosine metabolism pathway has the most metabolites.

Combining the results of the above enrichment analyses, the pathways of steroid hormone biosynthesis and tyrosine metabolism were further analyzed. Results showed that in positive-ion mode, the key metabolic pathways related to steroid hormone biosynthesis were oocyte meiosis, neuroactive ligand–receptor interaction, actin cytoskeleton regulation, cell adhesion molecule, and glycosaminoglycan-heparinsulfate-heparan sulfate biosynthesis. In addition, other important intermediates of metabolic network included 11-deoxycorticosterone, progesterone, 17α-hydroxyprogesterone, 11-deoxycortisol, acetylcholine, and 2-methoxyestrone (Fig. 8a). Further analysis showed that the abundances of 11-deoxycorticosterone, progesterone, 17α-hydroxyprogesterone, and 11-deoxycortisol were all indeed upregulated in the CCIS group compared with the CON group, indicating that the steroid hormone biosynthesis pathway was upregulated. Furthermore, results also showed that the upregulated acetylcholine might contributed to the upregulation of neuroactive ligand–receptor interaction and actin cytoskeleton regulation, while the downregulated 2-methoxyestrone might result in glycosaminoglycan-heparin/heparan sulfate biosynthesis to some extent (data not shown here). The analysis in the negative-ion mode found that succinic acid, gentisic acid, and 2, 5-dihydroxybenzoic acid esters were enriched in the tyrosine metabolic pathway, and the abundance of succinic acid in the CCIS group was significantly upregulated compared with that in the CON group (Fig. 8b). Therefore, it can be suggested that CCIS significantly changed the levels of progesterone, 17α-hydroxyprogesterone, hydrocortisone, 2-hydroxyestrone, succinic acid, gentisic acid, and 2-hydroxyprogesterone, which disturbed the metabolism of tyrosine and steroid hormone biosynthesis, leading to anti-growth performance and glycolipid metabolism disorder of the body. These results are helpful to explore potential biomarkers and therapeutic targets for the treatment of chronic stress of broilers and to provide guidelines for improving the performance.

### Correlation analysis of cecum microbiota, anti-growth performance, glycolipid metabolism disorder, and differential metabolites

**Fig 9:**
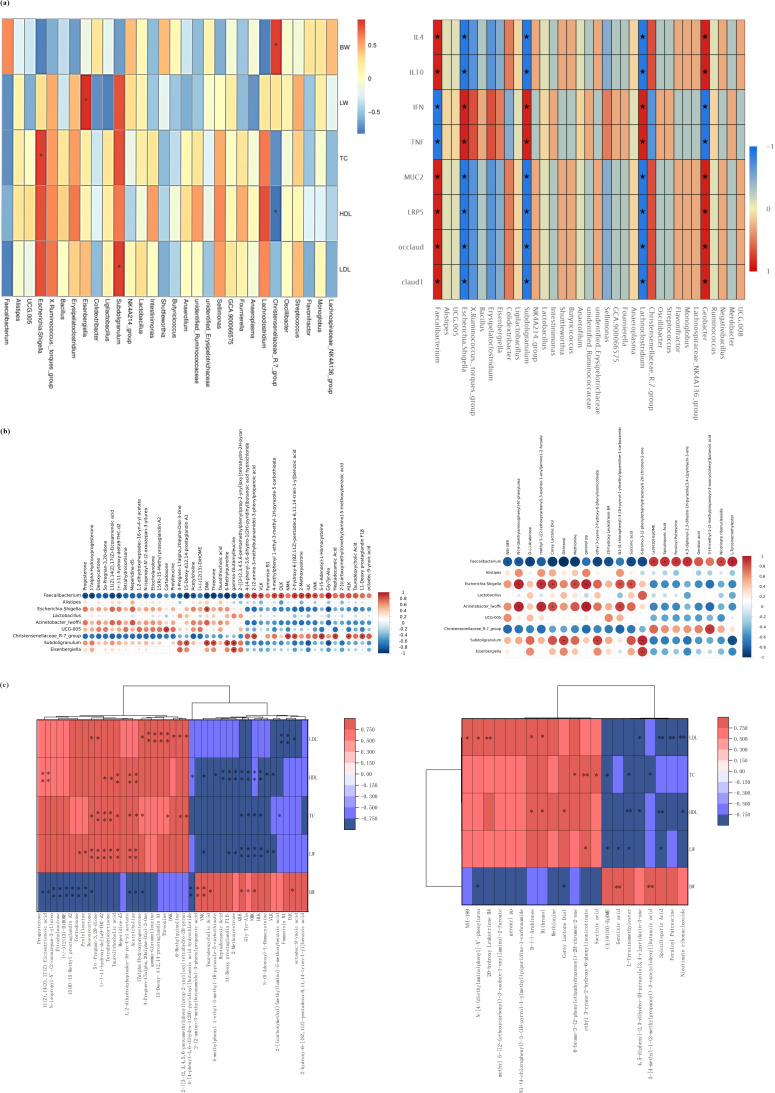
Correlation analysis among cecum microflora, anti-growth performance index, glycolipid metabolism disorder, and differential metabolites. (**a**) Correlation analysis between cecum microflora and glycolipid metabolism indexes. (**b**) Correlation analysis between differential metabolites and cecum microflora. (**c**) Correlation analysis between differential metabolites and glycolipid metabolism indexes.

In order to confirm whether the changed cecum microbiota resulted in anti-growth performance and glycolipid metabolism disorder in broilers, Spearman correlation analysis was used to further analyze the correlation between cecum microbiota and glycolipid metabolism parameters. The results showed that there was a significant positive correlation between BW and *Christensenellaceae_R.7_group*. In addition, liver weight (LW), blood TC, and LDLc were positively correlated with *Eisenbergiella*, *Escherichia_Shigella*, and *Subdoligranulum*, respectively, while HDLc was negatively correlated with *Christensenellaceae_R.7_group* (Fig. 9a).

To further explore the potential relevance of altered cecum microbiota and microbiota metabolites, based on the results of 16S rRNA sequencing, nine genera with high abundance and representative significance were selected, such as *Faecalibacterium*, *Alistipes*, *Escherichia_Shigella*, *Lactobacillus*, *Acinetobacter*, *UCG-005*, *Christensenellaceae*, *Subdoli granulum*, and *Eisenbergiella*; association analysis was performed with differential metabolites in the positive and negative modes, respectively. The results showed that the downregulated *Faecalibacterium* and *Christensenellaceae* in the CCIS group were negatively correlated with the upregulated differential metabolites and were positively correlated with the downregulated differential metabolites under the positive and negative-ion modes. However, *Alistipes*, *Escherichia_Shigella*, *Lactobacillus*, *Acinetobacter*, *UCG-005*, *Subdoligranulum*, and *Eisenbergiella* were positively correlated with the upregulated differential metabolites and were negatively correlated with the downregulated differential metabolites in the CCIS group. More specifically, in the positive-ion model, differential metabolites progesterone, 17α-hydroxyprogesterone, threonine, taurine cholic acid, 6-methylquinoline, and acetylcholine were negatively correlated with *Faecalibacterium*, while the correlations between *Escherichia_Shigella* and DNK, *Escherichia_Shigella* and Gly-Tyr-Ala, as well as *UCG-005* and CORT, were positively correlated. Furthermore, NSI-189, N-[4-(diethylamino)phenyl]-n'-phenylurea, D-(-)-arabinose, corey lactone diol, methionine, 20-hydroxy leukotriene B4, and succinic acid were negatively correlated with *Faecalibacterium*, while (±)9(10)-EpOME, spiculisporic acid, feruloyl putrescine, nicotinate ribonucleoside, and L-tyrosinemethylester were positively correlated with *Faecalibacterium*. In addition, *Escherichia_Shigella* was positively correlated with N-[4-(diethylamino)phenyl]-n'-phenylurea, corey lactone diol, methionine, geranyl-PP, and succinic acid. *Acinetobacter* was positively correlated with N-[4-(diethylamino)phenyl]-n'-phenylurea, corey lactone diol, and geranyl-PP. *Christensenellaceae* was positively correlated with NMK, heptadecanoic acid, and 11-deoxy prostaglandin F1β. *Subdoligranulum* was positively correlated with 15-deoxy-δ12, 14-prostaglandin a1, DNK, and threonine; and significantly negatively correlated with Gly-Try-Ala. Furthermore, *Eisenbergiella* has a significant positive correlation with γ-Glutamylleucine. These above results suggested that differential metabolites were indeed highly correlated with the cecum microbiota; the abundance of harmful bacteria was consistent with the abundance of differential metabolites upregulated by the CCIS group, while the abundance of beneficial bacteria was consistent with the abundance of differential metabolites downregulated by the CCIS group (Fig. 9b).

In addition, in the positive and negative-ion modes, differential metabolites were screened out and association analysis between them and BW, glycolipid metabolism disorder indexes were performed. The results showed that, in the positive-ion mode, the BW was negatively correlated with progesterone, desoxycortone, acetylcholine, cortodoxone, and prolylleucine, and positively correlated with fumonisin B1, HLK, NMK, and heptadecanoic acid; however, LW, TC, HDLc, and LDLc presented opposite results from BW. A more detailed results showed that LW was positively correlated with desoxycortone, cortodoxone, acetylcholine, and tetrahydrocortisone, while negatively correlated with heptadecanoic acid, 2-methoxyestrone, Gly-Tyr-Ala, and taurodeoxycholic acid. Furthermore, TC was positively correlated with progesterone, taurolithocholic acid, and acetylcholine. In addition, HDLc was negatively correlated with heptadecanoic acid, 2-methoxyestrone, Gly-Tyr-Ala, taurodeoxycholic acid, tetrahydrocortisone, taurolithocholic acid, 5α-pregnan-3,20-dione, acetylcholine, and VLK (Val-Leu-OH Kinase), while positively correlated with progesterone, tetrahydrocortisone, taurolithocholic acid, and acetylcholine. LDLc was positively correlated with γ-Glutamylleucine, threonine, 4-pregnen-17α, and 20α-diol-3-one, and were negatively correlated with VLK, fumonisin B1, and 2-[(carboxymethyl)amino]-5-methoxybenzoic acid. Similarly, in the negative mode, BW was negatively correlated with upregulated differential metabolites, while LW, TC, HDLc, and LDLc were positively correlated with upregulated differential metabolites. Therefore, these above results demonstrated that upregulated metabolites in the CCIS group significantly decreased the BW and increased the LW, TC, HDLc, and LDLc, which resulted in anti-growth performance and glycolipid metabolism disorder of broilers to some extent (Fig. 9c).

**Fig 10:**
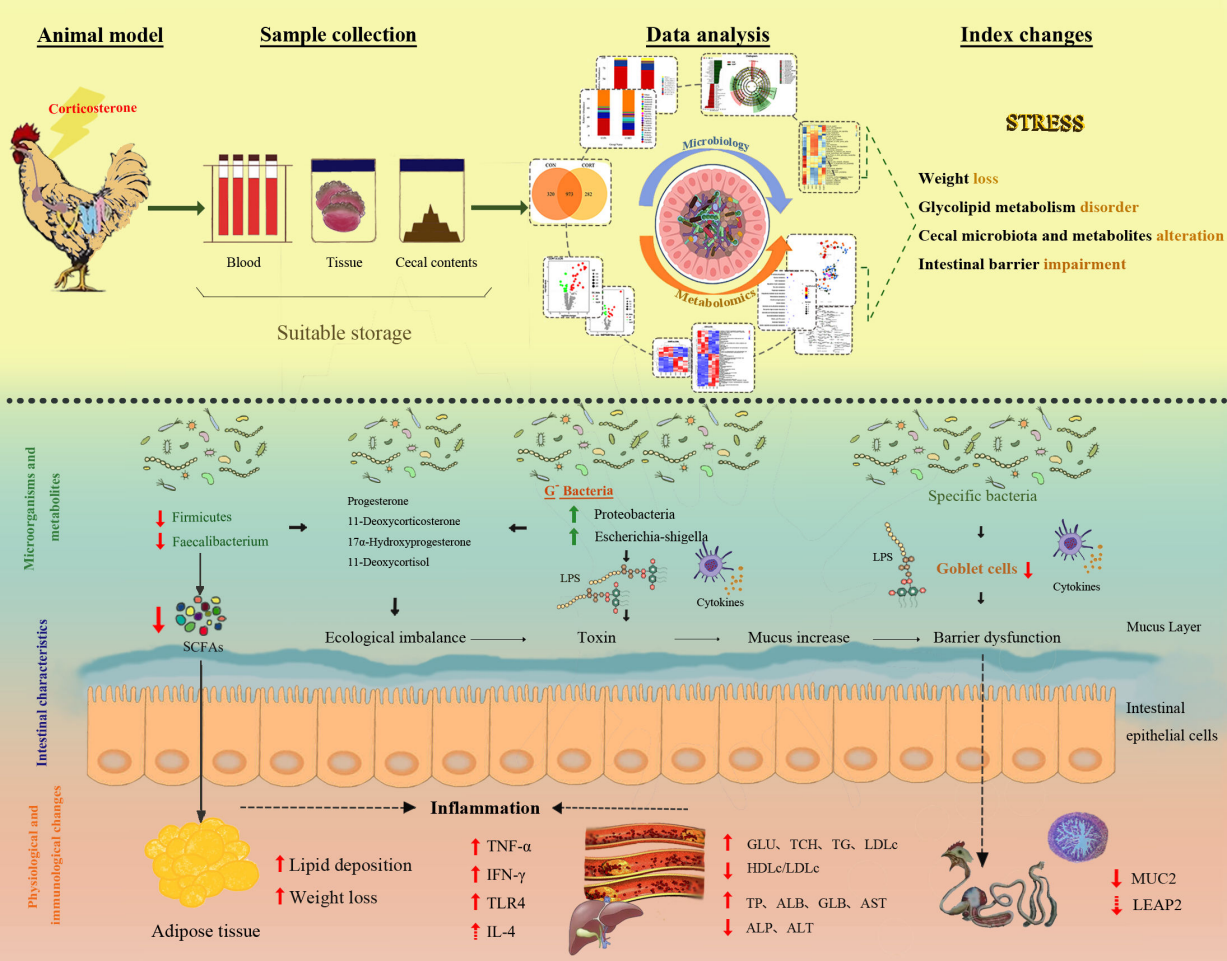
Summary of the regulation of altered cecum microflora and differential metabolites in anti-growth performance and glycolipid metabolism disorder in broilers under chronic stress exposure.

The regulation of cecum microbiota alterations and differential metabolites in anti-growth performance and glycolipid metabolism disorder in broilers under chronic stress exposure is summarized in Figure 10.

## DISCUSSION

China is the second largest country in chicken production, with about 4 billion yellow-feathered broilers listed on the market every year. Compared with other fast-growing broilers, yellow-feathered broilers have the characteristics of strong fat utilization ability and excellent meat quality (51), which have become the main source of chicken in China. In modern chicken breeding mode, one of the primary purposes of poultry enterprises is ensuring that the broilers attain market weight early (52); however, faster growth can put broilers under physiological stress, which makes broilers sensitive to diseases and affects growth performance, immunity, and intestinal health; therefore, maintaining gut health is indispensable for poultry industries. According to previous studies, exposing animals to a series of unknown mild stimuli for a long time is the most classic method to establish chronic stress model. This stress paradigm can avoid the adaptability of animals exposed to a single repeated stimulus. However, the model has poor repeatability and unity, and it is difficult to maintain the stress state for a long time (53, 54). In addition, lipopolysaccharide (LPS) injection are also used to establish inflammation-related chronic stress models (55, 56). However, this method can result in animal movement inhibition and some other side effects on the body (57). Currently, a growing number of studies have shown that the overactivity of HPA axis is an important pathophysiological manifestation of chronic stress. Persistent stress can lead to excessive hyperactivity of HPA axis, resulting in excessive ACTH and CORT (58, 59). Therefore, in this study, we established a chronic stress model by CORT injection, and this chronic stress model is established by directly disturbing the HPA axis, which has the advantages of long maintenance time and simple operation (60–62).

Previous studies have shown that chronic stress has a negative impact on growth promotion, appetite stimulation, immune enhancement, and antipathogen properties in livestock and poultry production (63–65). In the study, compared with the CON group, the FBW, BWG, and ADG of the CCIS broilers were decreased significantly, indicating that CCIS might lead to loss of appetite, digestive and absorptive capacity, resulting in a significant decrease in FBW, BWG, and ADG. This result was similar to that of previous studies. In addition, ADFI and F/G did not change in the CCIS broilers compared with the CON broilers, suggesting that CORT treatment had no change on feed conversion rate.

The basis of normal substance metabolism of animals and humans is good internal environment, which can be reflected by blood biochemical indexes. Plasma GLU, TC, TG, HDLc, and LDLc are important indexes indicating glycolipid metabolism capacity, and glycolipid metabolism disorder is characterized by the increase of the above indexes in the body. The present results showed that plasma GLU, TC, TG, HDLc, and LDLc were all significantly increased in the CCIS broilers compared with the CON broilers, while the HDLc/LDLc ratio was significantly decreased, suggesting that CCIS resulted in an increase in the incidence of glycolipid metabolism disorders. It is known that GLU is an important energy source for the body. Previous studies have shown that the decreased feed intake in broilers is related to the increase of blood GLU by CORT. Furthermore, as a key metabolic organ, liver plays a vital role in glycolipid metabolism, and its disorders may increase the risk of liver damage. The present results showed that CORT treatment significantly increased the contents of TP, ALB, and GLB, indicating that the synthesis capacity of body protein was enhanced. Furthermore, blood AST content and the AST/ALT ratio were both obviously increased, while ALP and ALT were significantly decreased, suggesting that CCIS-induced glycolipid metabolism disorders was closely related to liver damage in broilers. Studies have shown that chronic stress was able to increase the serum levels of NF-κB and TNF-α in rats, while the level of anti-inflammatory factor IL-10 and anti-apoptosis protein Bcl-2 was significantly decreased, indicating that chronic stress was able to upregulate inflammatory signals and promote apoptosis in rats (66). In addition, in alcohol-induced intestinal injury model, it was found that the participation of CORT was able to aggravate the injury of intestinal mucosal barrier and cause endotoxemia, systemic inflammation, and liver injury in mice, which was similar to our research results (1, 67).

Intestinal flora plays an important defensive role in numerous diseases, and most of the microbiota of broilers resides in the cecum. The cecum microbiota homeostasis is closely associated with enhanced nutrition absorption and increased growth performance. The current results showed that both the VH of ileum as well as the VCR of ileum and cecum were significantly increased, while the CD of jejunum and ileum was significantly reduced. Furthermore, the thickness of mucus layer of jejunum was significantly increased. These above results suggested that CCIS might affect intestinal development and function by changing the intestinal morphology. As a dynamic ecosystem, the structure of intestinal microbiota can be affected by numerous factors, among which stress is considered to be one of the most common factors. In this study, PCoA and PCA results showed that CCIS had a significant effect on the whole cecal microbial community structure. In terms of alpha diversity, the richness and diversity of cecum microbiota in the CCIS group were significantly higher than those in the CON group, indicating that CCIS resulted in a significantly different microbial diversity and richness, which decreased the intestinal immune function and the structure of cecum microbiota to favor weakened intestinal health in broilers.

According to the bar chart of the relative abundance of cecum microbiota of the CCIS and CON groups at the species level, we can directly observe the species with high relative abundance and the proportion of each sample at different classification levels. At the phylum level, CCIS significantly decreased the abundance of *Firmicutes* and increased the abundance of *Proteobacteria* in cecum. Previous studies have found that heat stress can decrease the number of *Firmicutes* and increase the number of *Proteobacteria* in the cecum of yellow-feathered broilers (68), which is consistent with our current results. In addition, similar results were obtained in the study investigating the effect of ginger juice on intestinal flora of LPS-induced acute lung injury mice, which showed that the relative abundance of *Proteus* (*Proteobacteria*) was significantly increased in the gut, while the relative abundance of *Firmicutes* and *Bacteroidetes* was significantly decreased (69, 70). Therefore, these above results demonstrated that CCIS was able to reduce the abundance of *Firmicutes* and *Bacteroidetes* and increase the *Proteobacteria* proportion, which led to the imbalance of microbial community composition and structure, and helped to obviously alleviate the damage of chronic stress to the body.

At the genus level, compared with the CON group, the abundance of *Faecalibacterium* was significantly decreased in the CCIS group, while *UCG-005* and *Escherichia_Shigella* were both significantly increased. *Faecalibacterium* is one of the most abundant bacteria in the gut, which not only coordinates the body against the invasion of foreign pathogens by producing butyrate, but also reduces the inflammatory response and improves the immune function by upregulating the expression of antibacterial peptides (71). *Faecalibacterium* is not only the main participant in the gut of humans and animals, but also the most important butyrate-producing bacteria in the gut, suggesting certain anti-inflammatory effects (72, 73). Butyrate has several beneficial properties that are essential to maintain gut health. Therefore, butyrate-producing bacteria can be seen as the next generation of probiotics, and *Faecalibacterium* can serve as a promising probiotic candidate for humans and animals suffering from chronic stress-related diseases. It has also been reported that *Faecalibacterium* can secrete microbial anti-inflammatory molecules and restores the structure and function of intestinal barrier by regulating the tight junction protein expression (33, 74). Furthermore, *Faecalibacterium* has high capacity in producing SCFAs, which play a key role in improving intestinal metabolic function, ameliorating various intestinal diseases (75), anxiety and depression (76, 77), and promoting stroke recovery in elderly mice (78). *Escherichia_Shigella* is a common intestinal pathogen, causing zoonotic diseases that endanger public health. It belongs to *Proteobacteria* and has a high abundance in various diseases (79–81). Taken together, in the study, CCIS significantly decreased the abundance of *Faecalibacterium* and increased the abundance of *Escherichia_Shigella* in the cecum of broilers, suggesting that CCIS could modulate intestinal health by reducing the abundance of intestinal beneficial flora and improving the abundance of harmful flora.

LEfSe analysis showed that 24 colonies such as *g_Lachnospira*, *g_Anaerostignum*, and *g_UCG_009* were enriched in the CON formation, while 23 colonies such as *f_Lachnospiraceae*, *o_Lachnospirales*, *p_Proteobacteria*, and *s_Acinetobacter_lwoffii* were enriched in the CCIS formation. *s_Acinetobacter_lwoffii* is a gram-negative aerobic bacteria; as an opportunistic pathogen, it endangers the immune system of the body (82). Previous studies have also shown that *s_Acinetobacter_lwoffii* has a damaging effect on both humans and animals, which is consistent with our current results and that *s_Acinetobacter_lwoffii* has a relative high abundance in the CCIS broilers. However, further studies are needed to determine whether it can be used as a potential microbial marker of broilers under chronic stress.

The functional classification of species information of cecum microbiota was analyzed by FAPROTAX software. It was found that the abundance of heterotrophic and fermented flora in the CCIS group was lower than that in the CON group, while the abundance of human pathogens, diarrhea pathogens, and nitrate reduction flora was increased, suggesting that CCIS could increase the number of pathogenic bacteria in broilers. Using Tax4Fun KEGG pathway annotation of intestinal microbial genes, we found that cecum microbiota in the CCIS group had significantly higher levels of functional genes related to translation, metabolism, prokaryotic flora community, nucleotide metabolism, and endocrine system. These above results showed that there were significant differences in cecum microbiota functions between the CON and CCIS broilers, indicating that CCIS could change the composition and structure of cecum microbiota, resulting in changes in intestinal environment and nutritional conditions, which directly or indirectly had adverse effects on various production performance. These results increased our understanding of the interaction between cecum microbiota and the host, and helped to resist chronic stress and improve the growth performance of broilers.

In the study, non-targeted metabolomics analysis was used to explore differential metabolites, and the association analysis among cecum microbiota, metabolites, and glycolipid metabolism indexes were further analyzed. A total of 710 positive model metabolites and 314 negative model metabolites were identified, of which lipids and lipid-like molecules accounted for the largest proportion. Furthermore, the contents of organic acids and their derivatives, organic oxides, and organic heterocyclic compound were also changed. Using VIP > 1.0, FC > 1.2 or FC < 0.833, and *P* value < 0.05 as the threshold, 43 differential metabolites were obtained, 24 of which were significantly increased, while 19 metabolites were decreased. In the negative-ion mode, 21 differential metabolites were significantly increased and eight metabolites were decreased. Based on differential analysis and KEGG pathway enrichment analysis, 18 secondary differential metabolic pathways were obtained, among which the global and overview maps were the most enriched, while others were amino acid metabolism, lipid metabolism, auxiliary factor and vitamin metabolism, carbohydrate metabolism, and nucleotide metabolism. The bubble chart of KEGG enrichment was drawn with the screening condition of *P* < 0.05 and FDR < 0.1. Finally, the most important four metabolic pathways were identified, including steroid hormone biosynthesis, steroid hormone metabolism, sulfur metabolism, and propanoate metabolism. Meanwhile, the metabolic pathway network diagram showed that progesterone, 17α-hydroxyprogesterone, hydrocortisone, 2-hydroxyestrone, succinic acid, gentistic acid, and 2-hydroxyprogesterone were enriched in the four metabolic pathways mentioned above. These above results are useful for exploring potential biomarkers against chronic stress. Furthermore, numerous studies have found that steroid hormone pathway plays a crucial role in various cellular functions as a transcriptional coregulation. Cholesterol is a precursor of steroid hormone biosynthesis and can be converted into steroid-related hormones (estrogen, androgens, glucocorticoids) or vitamin D, which are involved in glycolipid metabolism. In addition, previous studies *in vivo* showed that DEX (dexamethasone) injection significantly decreased the BW of rats, improved lipid deposition, and inhibited adipogenic gene expression, and decreased the richness and diversity of cecum microbiota (83). In broilers, it was also found that DEX could increase fat deposition by affecting hepatic AMPK (Adenosine 5‘-monophosphate (AMP)-activated protein kinase) expression and bile acid synthesis (84).

Next, the correlation between cecum microbiota and differential metabolites were further analyzed, which showed that the cecum microbiota was highly correlated with the differential metabolites; the upregulated metabolites were consistent with the abundance of *Alistipes*, *Escherichia_Shigella*, *Lactobacillus*, *Acinetobacter*, *UCG-005*, *Subdoligranulum*, and *Eisenbergiella*. Meanwhile, the association analysis of differential metabolites and glycolipid metabolism indexes also showed that the upregulated metabolites had a negative correlation with BW in the CCIS broilers, while LW, TC, HDLc, and LDLc presented opposite results from BW. According to the correlation analysis between cecum microbiota and glycolipid metabolism, it can be inferred that CCIS may regulate the cecum microbiota and differential metabolites, thus affecting growth performance and glycolipid metabolism. These above results demonstrated that CCIS significantly changed the composition and structure of cecum microbiota, and the production of bacterial metabolites, which might change the intestinal environment and nutritional condition, and directly or indirectly influence the production performance of broilers. These above findings increased our understanding of interactions among cecum microbiota, bacterial metabolites, and the host, and provided more ideas for both humans and animals to resist chronic stress and to increase growth performance of broilers in the future.

### Conclusion

Taken together, this study suggested that alterations of cecum microbiota and metabolites are involved in the development of CCIS, resulting in impaired growth performance and glycolipid metabolism disorder. To our knowledge, this is the first report that demonstrated that CCIS weakened the intestinal development, morphological structure, and biological functions of broilers through modulation of the microbial community, which seems to be adverse for gut health and growth performance.

## Data Availability

The data sets analyzed during the current study are available from the corresponding author upon reasonable request. The STORMS (Strengthening The Organization and Reporting of Microbiome Studies) checklist has been uploaded to the public data repository.
